# Deposition Characteristics of Air-Assisted Sprayer Based on Canopy Volume and Leaf Area of Orchard Trees

**DOI:** 10.3390/plants14020220

**Published:** 2025-01-14

**Authors:** Chenchen Gu, Jiahui Sun, Si Li, Shuo Yang, Wei Zou, Changyuan Zhai

**Affiliations:** 1 Intelligent Equipment Research Center, Beijing Academy of Agriculture and Forestry Sciences, Beijing 100097, China; gucc@nercita.org.cn (C.G.); sjiahui9@126.com (J.S.); lisi@nercita.org.cn (S.L.); yangshuo@nercita.org.cn (S.Y.); 2 Information Technology Research Center, Beijing Academy of agriculture and Forestry Sciences, Beijing 100097, China

**Keywords:** canopy volume, leaf area, air-assisted spraying, precision application, deposition characteristics

## Abstract

Precision pesticide application mainly relies on canopy volume, resulting in varied application effectiveness across different density areas of orchard trees. This study examined pesticide application effectiveness based on the spray wind, canopy volume, and leaf area within the canopy, providing variable bases for precise regulation of spray wind and pesticide dosage. The study addresses the knowledge gap by utilizing laser detection and ranging (LiDAR) to measure the thickness and leaf area of orchard tree canopies. The spray experiments were conducted on canopies of different regions, using an air-assisted sprayer with varying fan speeds of 1381 r/min, 1502 r/min, and 1676 r/min. The deposition effects were analyzed using water-sensitive papers. The inlet air speed within the canopy did not increase proportionally when the spray fan speed increased, and it showed a significant variation in locations with sparse foliage. Furthermore, droplets exhibited abnormal median volume diameters of the canopy regions with lower wind loss rates and smaller leaf areas. The influences were in the order of canopy thickness, leaf area, and inlet air speed on the cumulative deposition of droplets on both sides of the water-sensitive papers, as well as the ratio of deposition between the two sides, from big to small, are inlet air speed, leaf area, and canopy thickness. The study provides a scientific foundation for air control in precision pesticide application in apple orchards and contributes to the rapid development of precision spraying technologies.

## 1. Introduction

The control of orchard tree diseases can effectively improve the quality of fruit and increase farmers’ income [1,2,3,4]. Traditional disease control methods mainly rely on constant and undifferentiated pesticide application, resulting in pesticide waste and environmental pollution [5,6,7]. Air-assisted spray, recognized internationally, has been proven to effectively enhance pesticide utilization [8,9] and address the aforementioned issues in traditional spraying processes [10,11]. This technology consists of three main components: target detection, dosage regulation, and air control. Target feature detection is a prerequisite for the implementation of precision spraying technology, while dosage regulation and air control are the key steps in the execution of precise dosage spraying technology, serving as the crucial indicators for the accuracy of canopy detection technology.

Air can promote leaf disturbance, enhance the penetration capability of droplets through the canopy, increase pesticide deposition by about 30%, and reduce droplet drift by 75% [12]. Proper air speed in precision spraying can ensure uniform deposition of droplets on the surface and inner leaves of the canopy, achieving effective pesticide utilization and minimizing droplet drift.

Canopy volume was studied in the early research. Yu et al. investigated the canopy slicing technique for volume algorithms. Extract the surface and line information from the canopy point cloud, and the information was accumulated to obtain the canopy volume [13]. Dou et al. developed a variable spraying control system based on canopy volume algorithms for orchard trees [14]. The experimental results showed that the droplet deposition density was greater than 20 droplets/cm^2^, the droplet coverage rate was less than 30%, and the pesticide usage was reduced by 62.25%. Li et al. designed a profiling air-assisted sprayer that applied pesticides based on canopy volume [15]. By adjusting spray parameters in real time according to the canopy volume, the uniformity of droplet deposition in the canopy was improved, resulting in a 45.7% reduction in fluid usage and a 23.2% decrease in droplet drift. Throughout the 1-year growth cycle of orchard trees, the change in canopy volume is relatively small, while the density within the canopy undergoes significant fluctuations [16]. Precision pesticide application research primarily focuses on the effectiveness based on canopy volume, leading to severe waste of pesticides in sparse foliage areas and inadequate application in dense foliage areas of orchard trees, which ultimately affects the overall effectiveness of pesticide application.

In recent years, the pesticide application technology based on canopy density has received great attention. The leaf area index (LAI), leaf area density (LAD), and canopy leaf area (LA) are used to characterize canopy density. LAI represents the leaf area per unit area in the vertical direction, which is more suitable for aerial spraying [17,18]. LAD reflects the leaf area per unit volume [19,20], but its calculation method is complex and cannot meet the real-time operational requirements of variable-rate spraying. Sanz et al., Zhang et al., and Berk et al. have studied detection models for canopy leaf area in simulated trees and trees with thin canopies [21,22,23]. The models they established are simple, but their reliability needs further verification. Gu et al. focused on orchard trees with thick canopies, conducted research on the detection model of leaf area, and established an advanced algorithm for canopy leaf area detection [16]. Li et al. studied methods for assessing canopy leaf area using light detection and ranging (LiDAR) multiple returns [24], and the results showed that the TGA method is more suitable for spraying. While the above-mentioned studies have improved the accuracy of model calculations, they have not conducted pesticide application experiments in orchards. The actual effectiveness is unknown. Studies based on canopy density mainly focus on detection methods and model research, with less emphasis on studying the droplet deposition characteristics. In air-assisted spraying, the deposition characteristics of droplets within the canopy are not only affected by wind force but also by canopy thickness and leaf density within the canopy.

Currently, research on precision pesticide application technology primarily focuses on single factors, lacking comprehensive studies targeting the key factors that influence the effectiveness of pesticide application in orchard trees [25,26,27,28]. Wind speed, canopy volume, and leaf area within the canopy are crucial factors affecting application effectiveness. To study the influence of the three factors, LiDAR is used to detect the canopy thickness and leaf area, based on the previous study on previous canopy volume detection [29] and the fitting model of LiDAR point cloud data and the leaf area [16]. Different spray fan speeds of 1381 r/min, 1502 r/min, and 1676 r/min are set on the sprayer. The sum of deposition on the front and back of the paper, as well as the ratio of deposition on the front and back, are used as indicators to study the characteristics of droplet deposition distribution in relation to canopy thickness, leaf area, and air speed. This study on application effectiveness based on the comprehensive factors can provide a variable basis for precise regulation of both wind force and pesticide dosage, enabling their coordinated control. This will enhance pesticide utilization efficiency and drive the development of precision pesticide application technology.

## 2. Results

According to the spraying machine operation method, the distribution of droplet deposition on both sides of the water-sensitive paper after a single spray can be used as an indication of the deposition situation on the leaves of orchard trees after completion of the spraying operation on both sides. This study investigates the relationship and models between the influencing factors (independent variables), such as canopy inlet air speed, canopy thickness, and canopy leaf area, and the dependent variables, such as droplet VMD, droplet deposition on the front and back of water-sensitive paper, and the ratio of droplet deposition on the front and back.

### 2.1. Deposition of Droplets on Water-Sensitive Paper

**Figure 1:**
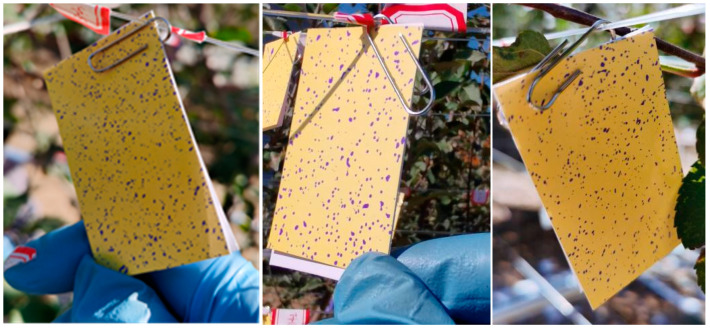
Droplet deposition on water-sensitive paper.

Spraying machine operations are conducted on both sides of the orchard trees in the inter-row spaces, with one side of the trees being sprayed and then switching to the other side after completion of the operation. Data from the front and back sides of the water-sensitive paper is considered equivalent to the results obtained from spraying both sides of the canopy. This study is based on the experimental data of droplet deposition on both sides of the water-sensitive paper in the deposition test. The results of droplet deposition on the water-sensitive paper after the experiment are shown in Figure 1.

**Figure 2:**
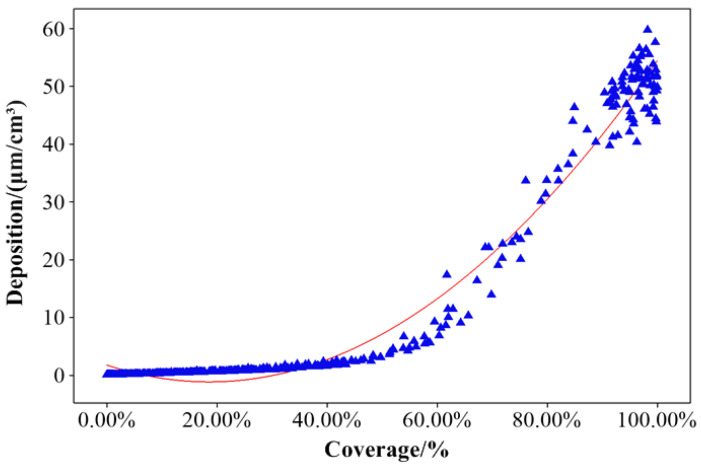
Correlation analysis between droplet coverage and deposition on water-sensitive paper.

The main indicators for measuring the deposition effectiveness of droplets within the canopy include droplet VMD, deposition density, coverage ratio, and deposition amount. To further optimize the research subjects, a study was conducted to investigate the relationship between droplet coverage and deposition (Figure 2). The study found that a quadratic correlation between the two was relatively significant.

A quadratic regression analysis was conducted to fit the data, resulting in the regression equation (Equation (1)). The regression analysis yielded an R^2^ value of 0.981, with a *p*-value of 0, indicating a significant fit for the regression equation. Therefore, either of the two reference indicators can be selected as the research indicator.

(1) $$D=1.747-31.32C+84.07C^{2}$$

where:

*D*—deposition amount, μL/cm^3^;

*C*—coverage ratio, %.

### 2.2. Effects of Different Inlet Air Speeds in the Canopy on the VMD of Droplet Deposition

**Figure 3:**
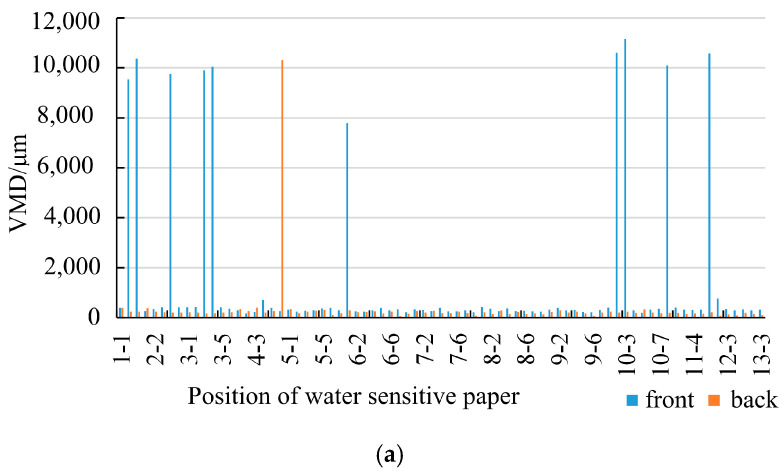

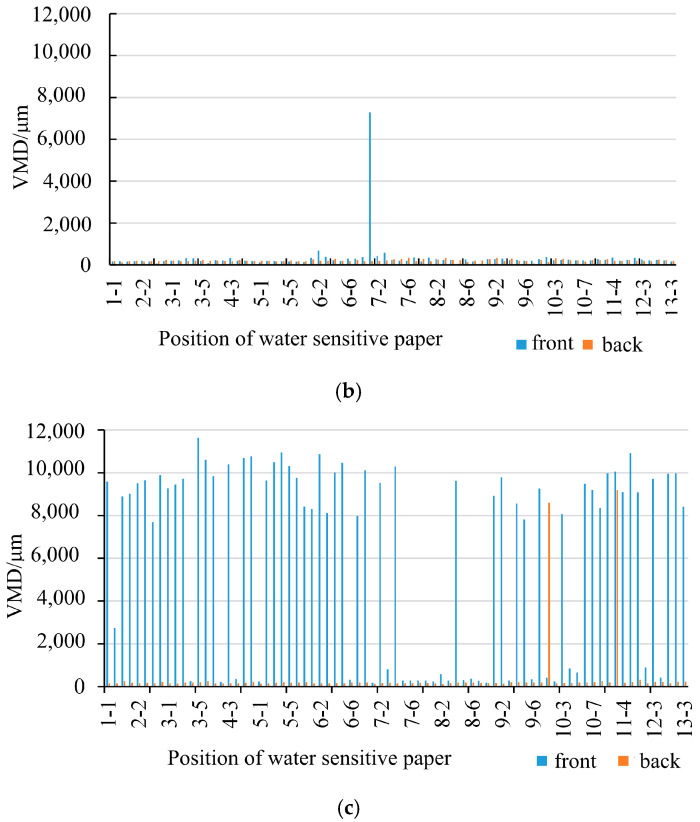
VMD value of droplet deposition in the canopy in different areas of different spray fan speeds. (**a**) Spray fan speed of 1381 r/min. (**b**) Spray fan speed of 1502 r/min. (**c**) Spray fan speed of 1676 r/min.

The VMD is a crucial parameter reflecting droplet size, with smaller values indicating finer droplets and larger values indicating larger droplets. JB/T 7875-1999 (1999) divides droplets into four categories based on VMD: smoke (VMD < 50 μm), mist (50 μm ≤ VMD ≤ 100 μm), fine mist (100 μm ≤ VMD ≤ 400 μm), and coarse mist (VMD > 400 μm) [30]. Figure 3 shows the VMD of droplets deposited on water-sensitive paper placed on the front and reverse sides under different fan speeds within the canopy.

**Figure 4:**
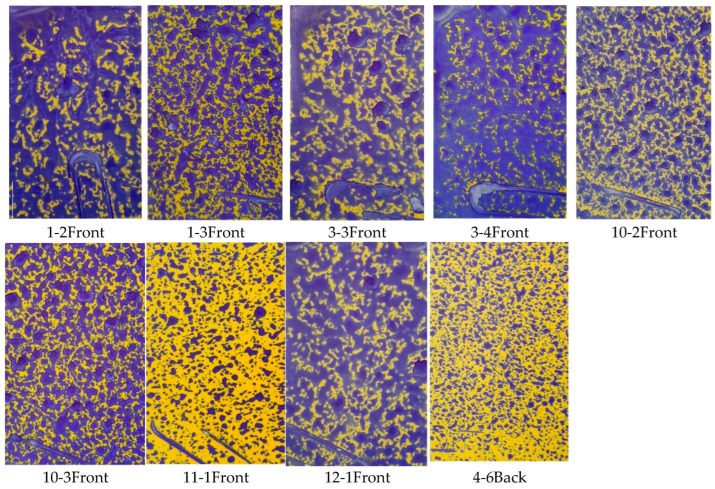
Water-sensitive paper with large VMD values (Figure 7a) under the condition of a spray fan speed of 1381 r/min.

**Figure 5:**
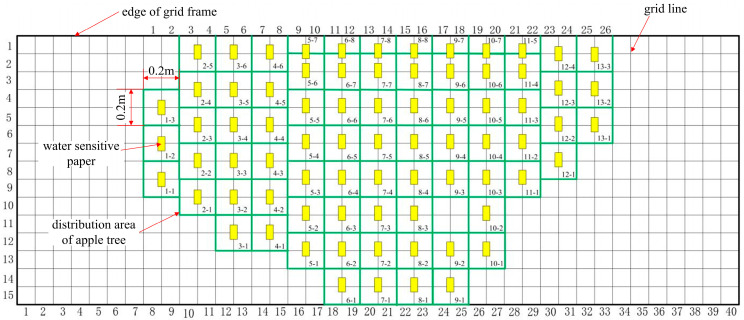
Schematic diagram of the water-sensitive paper’s fixed position in the canopy.

**Figure 6:**
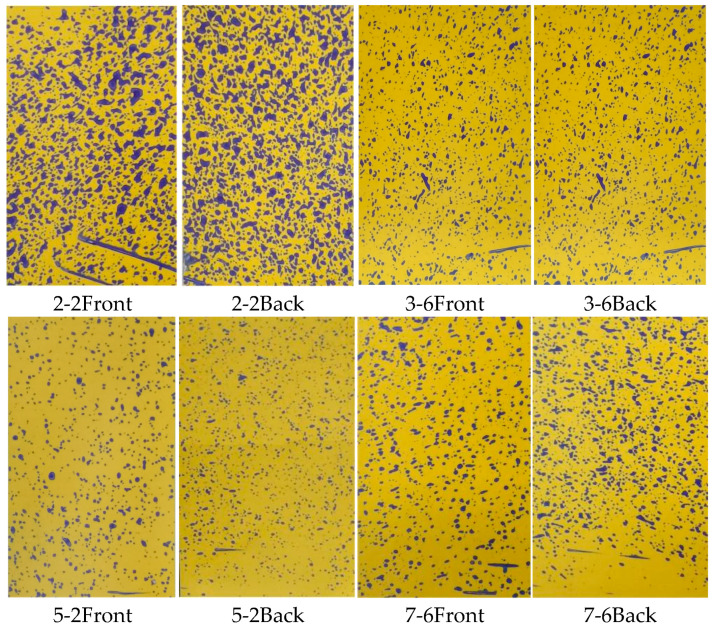
Water-sensitive paper with normal VMD values under the condition of a spray fan speed of 1381 r/min.

The larger the VMD value, the coarser the droplets and the larger the droplet size. Figure 3a–c illustrate the VMD values of water-sensitive paper at different locations under the operation of the sprayer with fan speeds of 1381 r/min, 1502 r/min, and 1676 r/min, respectively. Figure 3a, the VMD values are generally low, but there are a few groups with relatively high VMD values. Figure 3b shows better results with almost no abnormal VMD values, while Figure 3c exhibits a higher occurrence of abnormal VMD values. The VMD of droplet deposition on the water-sensitive paper is correlated with canopy thickness, leaf area, and inlet air speed. Upon observation of the water-sensitive paper corresponding to the group with high VMD values (Figure 4), it is apparent that the deposition of droplets on the paper is substantial, leading to a higher droplet coverage and larger droplet sizes. Locations with larger VMD values (Figure 3a) correspond to canopy features and input airflow speed conditions, as shown in Figure 5. The air speed, air speed loss rate, canopy thickness, and leaf area of these areas are presented in Table 1. Comparing with Figure 6 (water-sensitive paper corresponding to normal VMD values) and Table 2 (locations with smaller VMD values correspond to canopy features and input airflow speed conditions), it is evident that the wind loss in the abnormal VMD group is lower than that in the normal group, with a smaller leaf area within the canopy. These positions are typically located at the edge of the canopy, characterized by a thinner canopy and less leaf distribution. During the spraying process, the air speed at different locations within the canopy varies significantly with the same spray quantity. The droplets travel a shorter distance within the canopy, experiencing less resistance from the foliage. As a result, the water-sensitive paper receives a greater amount of droplets.

**Figure 7:**
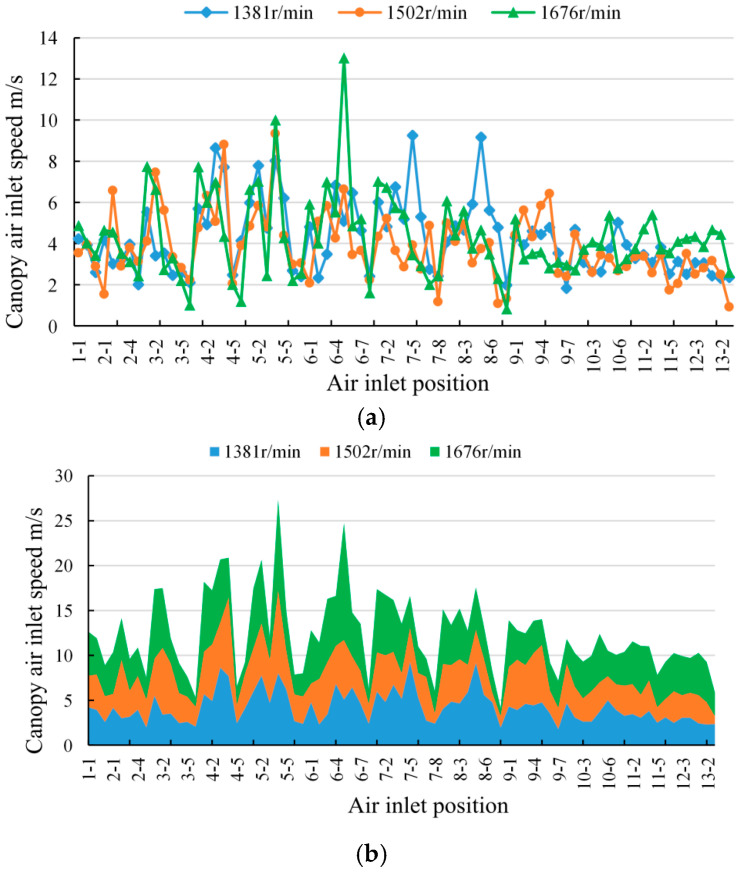
Airflow speed at canopy inlet under different fan speed conditions. (**a**) Line diagram of airflow speed at canopy inlet under different fan speed conditions. (**b**) Stacked area chart of air speed at canopy inlet under different fan speed conditions.

Since the spray volume was set as a constant in this study (non-ultra-low-volume spraying), the deposition of droplets on the front side of the water-sensitive paper is generally larger, leading to the occurrence of abnormally high VMD values. The deposition of droplets on the back side of the water-sensitive paper is more uniform, with only one instance of a significantly larger deposition (position 4–6). In this particular area, the wind loss rate is high, the inlet air speed into the canopy is large, and the canopy thickness is substantial. Most probably, the leaf area within the canopy is relatively sparse, allowing the wind force to carry the droplets around the water-sensitive paper and deposit them on the backside. Alternatively, during the spraying process, the wind force causes the water-sensitive paper to flip over, resulting in the droplets coincidentally depositing on the flipped back side. After the spraying, the water-sensitive paper reverts to its original position without the influence of wind force. It can be concluded from Figure 3a–c that higher spray fan speed does not necessarily lead to a better deposition effect. The reason is analyzed in Figure 7.

Comparison of inlet air speeds at the canopy entry under different fan speed conditions for the same canopy thickness and leaf area distribution. Figure 7a,b present the canopy entry air speeds under various sprayer fan speeds. Figure 7a illustrates the line graph depicting the air speeds at the canopy entry under different air conditions, while Figure 7b displays the cumulative area plot of the canopy entry. The line graph provides insights into the trends, fluctuations, and magnitude of the data, while the cumulative area plot allows for an understanding of the trends, overall data scales, and the proportions of different data items. Based on Figure 7a, it can be observed that the air speeds at the canopy entry fluctuate significantly within the range of 2 m/s to 8 m/s under different conditions. Figure 7b reveals that the air speed is highest for the fan speed of 1676 r/min, while it is relatively lower for the fan speed of 1502 r/min.

From the above analysis, it can be concluded that increasing the air speed at the canopy entry enhances the deposition of droplets within the canopy. A higher air speed at the entry results in larger droplet diameters on the water-sensitive paper. Moreover, the increase in canopy entry air speed is not proportional to the increase in sprayer fan speed, indicating a non-linear relationship between the air speed at the spraying machine outlet and the fan speed.

Figure 7 displays the measurements of the canopy entry air speeds at different positions under various fan speed conditions. It can be observed that there are significant differences in the air speed differentials at positions 3-1, 4-1, 5-4, and 6-5. Referring to the positions of the water-sensitive paper within the canopy as shown in Figure 5, positions 3-1 and 4-1 are located in the lower part of the canopy and are closer to the outlet of the spraying machine fan, resulting in larger fluctuations in air speed with different fan speeds. Positions 5-4 and 6-5 are situated in the middle of the canopy, farther away from the upper outlet of the spraying machine, and have larger canopy leaf area values, leading to increased wind resistance.

After the wind is emitted from the outlet, it travels a certain distance before reaching these positions. The wind attenuation varies significantly for different fan outlet air speeds over the same distance. Due to the larger canopy leaf area values, the wind encounters greater resistance as it passes through the canopy. Positions 3-6, 4-6, and 8-8 exhibit smaller differences in canopy entry air speeds. Upon observation of Figure 5, it can be noted that these positions are located at the highest part of the canopy, farthest from the canopy outlet, and possess canopy leaf area values around 0.9 cm^2^, which are relatively sparse. Consequently, air speed variations are minimal in these positions.

### 2.3. The Effects of Canopy Characteristics and Air Speed on the Deposition of Droplets on the Leaf Surfaces

In this study, a total of 231 sets of experimental data were obtained by conducting three experiments with different fan speeds, each providing 77 sets of data on water-sensitive papers. Out of these, 119 sets of valid droplet deposition data were extracted by analyzing the effective droplet deposition. Correlation analysis was employed to investigate the relationship between canopy inlet air speed, canopy thickness, canopy leaf area, and the total deposition of airborne droplets on the adaxial and abaxial sides of water-sensitive papers.

**Figure 8:**
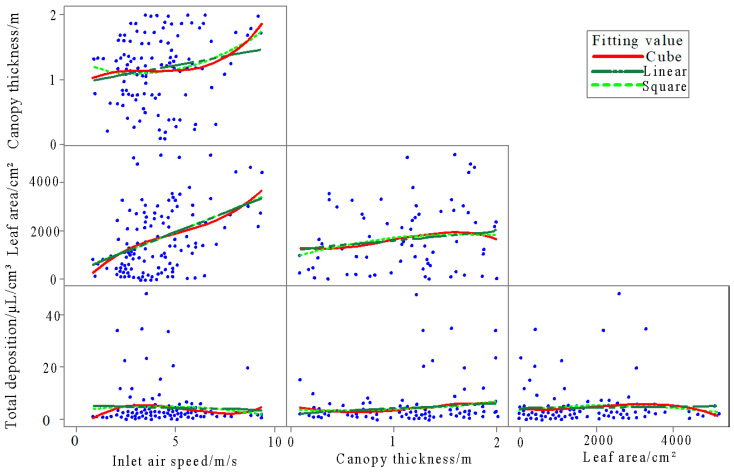
Matrix diagram of the relationship between inlet airflow speed, canopy thickness, leaf area, and total deposition.

Figure 8 presents the correlation between the influencing factors and the total deposition of droplets on water-sensitive papers. Linear, quadratic, and cubic curve fittings were applied, and it was observed that the level of data dispersion among inlet air speed, canopy thickness, and leaf area in relation to total deposition was relatively similar. Furthermore, the three factors showed significant data dispersion between each other, indicating minimal influence between them. The curve fitting results did not significantly vary with different fitting orders due to the inherent data dispersion. The Pearson correlation analysis demonstrated the correlations between inlet air speed, canopy thickness, leaf area, and total droplet deposition (Table 3).

According to Table 3, the correlations between inlet air speed, canopy thickness, leaf area, and total deposition are 0.045, 0.128, and 0.023, respectively. It is evident that the correlations between these factors are relatively weak, indicating minimal influence between them. Specifically, the correlations between inlet air speed and leaf area, as well as between canopy thickness and leaf area, are also weak, and no quadratic interactions are present.

**Figure 9:**
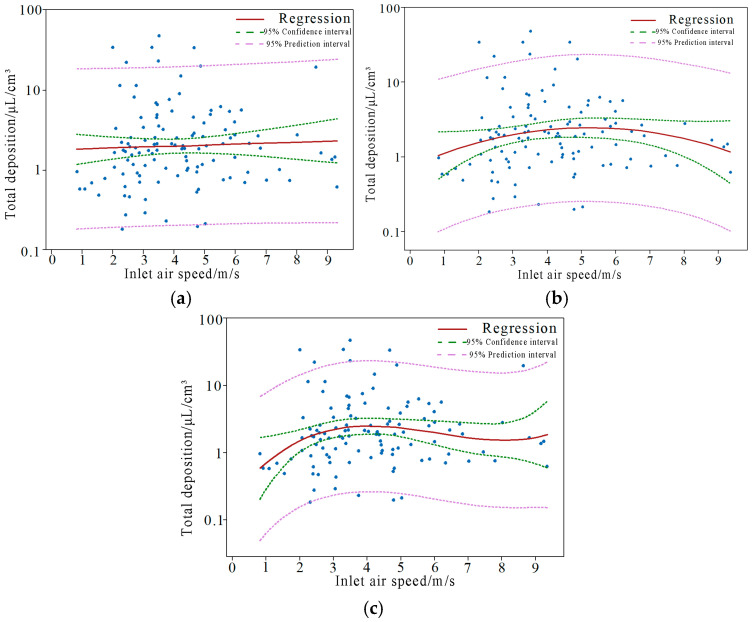
Curve of three times fitting relationship between inlet airflow speed and total droplet deposition. (**a**) Linear fitting curve graph. (**b**) Quadratic fitting curve graph. (**c**) Cubic fitting curve graph.

Figure 9 illustrates the results of the linear, quadratic, and cubic curve fittings between the inlet air speed and the total deposition. The prediction interval represents the range of predicted values for the variable. By utilizing the estimated prediction equation, we can determine the estimated interval for the dependent variable y for a given value x’0 of the independent variable x’. The confidence interval reflects the estimation of parameters. By using the estimated regression equation, we can calculate the average estimated interval for the dependent variable y’1 for a given value x’1 of the independent variable x’, which reflects the uncertainty of the predicted mean.

From Figure 9, it can be observed that the three function equations, derived from the boundary lines of the prediction interval, fail to completely predict the distribution range of the deposition total. This suggests that the function equations obtained from the data fitting results cannot accurately predict the actual distribution range of the data. The prediction interval is consistently wider than the confidence interval, with narrower intervals when x’ is closer to the mean and wider intervals when x’ is further from the mean. In practical applications, narrower confidence intervals are preferred. In Figure 9, the confidence interval becomes narrower as the degree of curve fitting increases, but the overall change is minimal.

Research was conducted on the linear, quadratic, and cubic fittings of inlet air speed, canopy thickness, and leaf area to the total deposition. The overall comparative analysis of the obtained R^2^ values for the fitted equations is presented in Table 4. The max value of R^2^ is 0.05, which shows there were weak correlations of inlet air speed, canopy thickness, leaf area, and total droplet deposition. It can be observed that all R^2^ values are relatively low, gradually increasing with an increase in the degree of fitting. Specifically, the R^2^ values for the quadratic and cubic fitting of the inlet air speed are significantly higher than the other two methods, while the linear fitting shows slightly lower effectiveness. This suggests that the influence of the inlet air speed on the total deposition of water-sensitive paper droplets is greater compared to canopy thickness and leaf area. The influence was obtained by ordering the R^2^ values. From greatest to least, the influence is as follows: inlet air speed, leaf area, and canopy thickness.

Table 5 presents the significance analysis of obtaining the fitted equation. A smaller *p*-value indicates a better significance of the obtained equation. From the table, it can be observed that a higher order of fitting does not necessarily lead to a better fit to the data. The *p*-values at the third-degree polynomial fitting are all greater than the *p*-value of the second-degree polynomial fit. After the second-degree fitting, the *p*-values for both the entrance air speed and canopy thickness are smaller compared to the *p*-value of the linear fit, indicating an improved accuracy of the equation. In terms of the influence of leaf area on the total deposition, the *p*-value is the smallest for the linear fit, and it increases with the increase in the order of fitting. This suggests that the relationship between leaf area and the total deposition is better suited to a linear relationship, while the other two factors are better suited to a second-degree fit. None of the three factors are suitable for third-degree fitting.

To further compare the data, regression equations were fitted for the relationships between inlet air speed, canopy thickness, leaf area, and total deposition based on the analysis above. Based on Figure 6, it can be concluded that there is a relatively weak correlation between the dependent variables and no interaction among the data. After fitting the linear polynomial regression equations, it was found that the second-degree fitting for entrance air speed and canopy thickness and the first-degree fitting for leaf area produced better results. In the process of determining the regression equation, there were no interaction terms between the independent variables, but the second-degree terms for entrance air speed and canopy thickness were included, resulting in the fitted regression Equation (2).

(2) $$D_{T}\;=\;2.64+0.76C_{1}-2.54C_{2}+3.06\times 10^{-4}C_{3}-0.128C_{1}^{2}+2.16C_{2}^{2}$$

where:

*D*_T_—total deposition, μL/cm^3^;

*C*_1_—inlet air speed, m/s;

*C*_2_—canopy thickness, m;

*C*_3_—leaf area, m^2^.

Table 6 shows the *p* values obtained through analysis of variance for each data item. By observing the *p* values, it can be concluded that the *p* value for the fitted equation is 0.531, which is greater than 0.1, indicating that the model is not significant. After analyzing the various variables, it was found that all of them have *p* values greater than 0.1, indicating that none of the variables significantly contribute to the fit of the equation. Furthermore, the R^2^ value of the fitted equation is 0.004, suggesting weak explanatory power of the equation for the data.

Through the study, it has been discovered that there is no significant correlation between canopy inlet air speed, canopy thickness, leaf area, and the total deposition of droplets on both sides of the sensitive paper. Previous research has identified canopy inlet air speed, canopy thickness, and leaf area as factors influencing spray effectiveness. The reasons for the lack of a significant relationship can be attributed to the initial high velocity of droplets atomized by the nozzle, which reduces the impact of inlet air speed and canopy density on spray efficiency. Additionally, a low inlet air speed does not assist in the movement of droplets within the canopy, leading to deposition at their original velocity. The selected experimental canopy has a sparse distribution of leaves, allowing droplets to penetrate through the canopy without obstruction from the leaves.

### 2.4. The Influence of Canopy Characteristics and Air Speed on the Deposition Ratio of Droplets on Leaf Surfaces

The air can disturb the leaves during the process of spray, thereby increasing the deposition of droplets on the back of the leaves. In this study, the deposition ratio of droplets on the front and back sides of leaves is proposed as a measure of the deposition effect of the inlet air speed. The method described in Section 2.2 was used to extract valid deposition data from the water-sensitive paper samples by removing any unreasonable deposits. The study was conducted using the inlet air speed, canopy thickness, and leaf area as independent variables and the ratio of the deposition of droplets on the front and back sides of the water-sensitive paper as the dependent variable.

**Figure 10:**
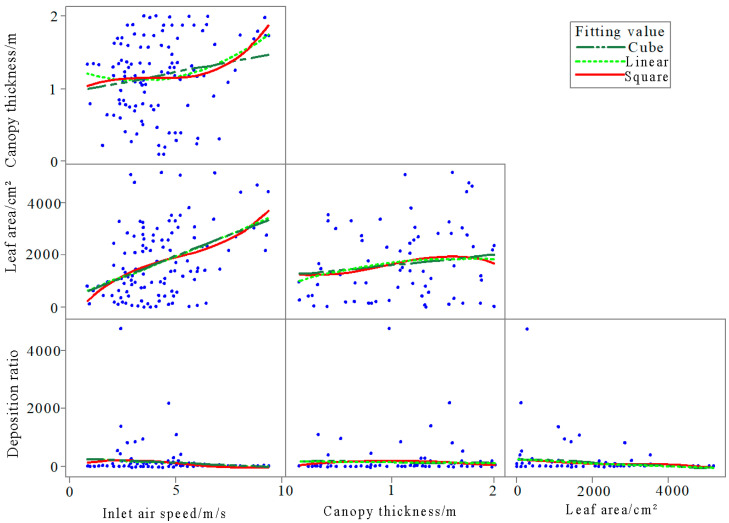
Correlation diagram of inlet air speed, canopy thickness, and leaf area with deposition ratio.

Based on the analysis from Figure 10, it can be concluded that the three influencing factors have similar correlations with the deposition ratio of droplets on both sides of the water-sensitive paper. The differences in linear, quadratic, and cubic fitted lines for the data are not significant.

Based on the linear, quadratic, and cubic fitting of the relationship between inlet air speed, canopy thickness, and leaf area with the deposition ratio of droplets on the front and back side of the water-sensitive paper, a comparative analysis was conducted to obtain the R^2^ values of the fitted equations (Table 7). It can be observed from the graph that the cubic fitting results in lower R^2^ values for the data. As the fitting order increases, the R^2^ values for all three factors increase. The R^2^ value for the inlet air speed shows greater fluctuations, while the R^2^ values for the inlet air speed and canopy thickness tend to increase with an increasing number of iterations. On the other hand, the R^2^ value for the canopy leaf area increases at a slower rate or even decreases as the number of iterations increases. The impact sequence of these three factors on droplet deposition is as follows from large to small: inlet air speed, leaf area, and canopy thickness. This result is the same as the findings on the overall deposition influenced by inlet air speed, canopy thickness, and leaf area.

(3) $$D_{R}=-179+257C_{1}+389C_{2}-0.216C_{3}-57.6C_{1}^{2}-180C_{2}^{2}+6.8\times 10^{-5}C_{3}^{2}+3.65C_{1}^{3}$$

where:

*D*_R_—deposition ratio.

Equation (3) represents the regression equation obtained through multiple regression analysis, where the regression process includes a cubic fitting term for the leaf area. The calculated coefficient for the cubic fitting term of the leaf area in the equation is 0, indicating that the linear term of the leaf area is the significant term for the data, which slightly deviates from the judgment based on the *p* value of the fitted equation (Table 8). The *p* value for linear fitting of the leaf area is similar to that of the cubic fitting, and both are smaller than the *p* value for quadratic fitting, suggesting that both linear and cubic fittings could potentially be significant terms.

Table 9 displays the *p* values for the variance analysis of each data item. By observing the *p* values, it is found that the *p* value for the fitted equation is 0.744, which is greater than 0.1, indicating that the model is not significant. Analysis of the individual correlation terms reveals that all of them are greater than 0.1, indicating that none of the data items significantly contribute to the equation fitting. The R^2^ value for the fitted equation is 0.004, indicating a weak explanatory power of the equation for the data. Through analysis, it is concluded that the independent variables of canopy inlet air speed, canopy thickness, and leaf area have a weak impact on the deposition ratio of droplets on both sides of the water-sensitive paper in this study. There is no significant correlation among these variables, leading to poor significance of the obtained regression model.

In this experimental process, the study focused on commonly used spray rates during the operation of the sprayer. The variation in droplet deposition within the canopy under different spray rates and airflow conditions was not investigated. Further discussion and research are needed to explore the issue of poor significance in the regression model, and it is suggested as an important future direction for in-depth study.

## 3. Materials and Methods

### 3.1. Orchard Detection Object

**Figure 11:**
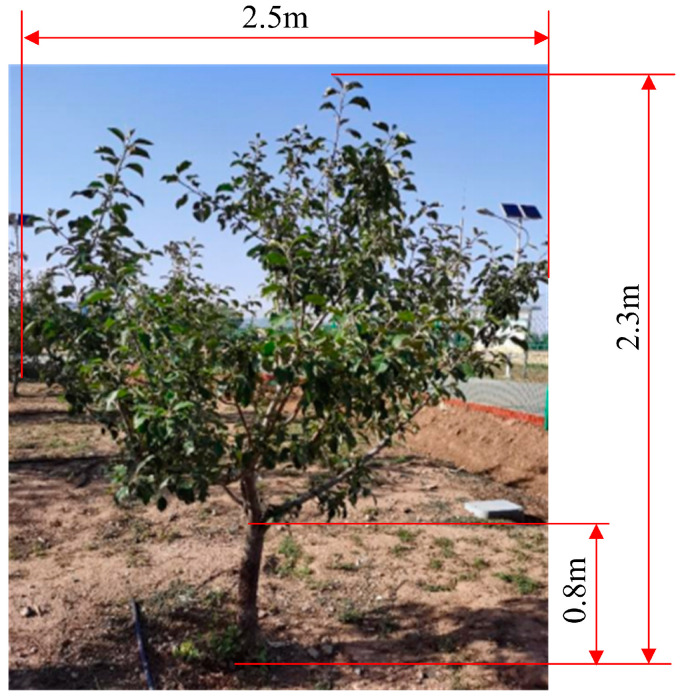
Orchard tree for drop deposition characteristic test.

The experiment was conducted on orchard apple trees, as shown in Figure 11, during the growing season of principal growth stage 8: maturity of fruit and seed [31]. According to the definition of growth stages for pome fruit in “Growth stages of mono-and dicotyledonous plants”, the orchard trees were in the last stage 81: beginning of ripening: first appearance of cultivar-specific color [31]. At this stage, the fruit began to ripen, exhibiting the specific color of the cultivar and possessing the typical flavor and hardness. The growth of apple tree leaves almost ceased; there was no leaf shedding, and there were no apparent changes in the leaves within the canopy. The test site was the Xiaotangshan National Precision Agriculture Research Demonstration Base in Changping District, Beijing. The tree species was the Fuji apple, and the trees were 5 years old. The shape of the tree was an open center, which had an obvious middle trunk, main branch, and natural stratification. The size of the tree was 2.3 m in height and 2.5 m in width. The row spacing was 4 m, and the plant spacing was 3.5 m. The wind speed was measured nearby in the orchard. The max wind speed was less than 1 m/s, which rarely influenced the experimental apple tree canopy.

### 3.2. Water-Sensitive Paper Layout

**Figure 12:**
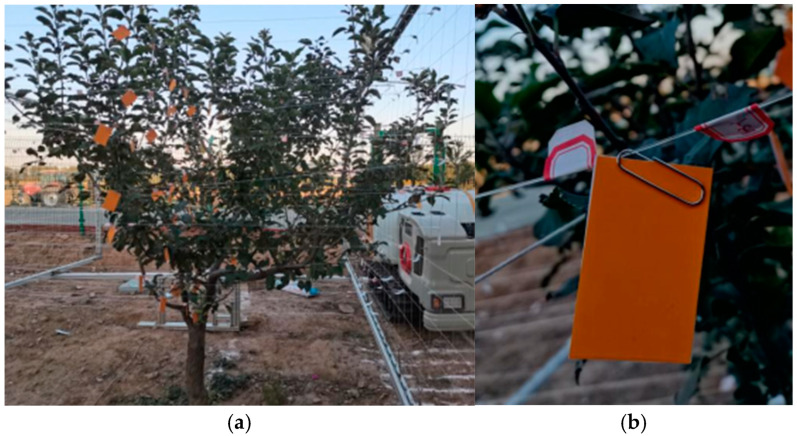
Layout and arrangement of water-sensitive paper in the canopy. (**a**) Water-sensitive paper layout in the canopy. (**b**) Water-sensitive paper arrangement.

This study utilized a three-dimensional measurement platform [8] to locate and partition the canopy of orchard trees, facilitating the arrangement of water-sensitive paper. The arrangement of the water-sensitive paper is shown in Figure 5, with a measurement area size of 0.2 m × 0.2 m. The yellow cuboid in the diagram represents the position of the water-sensitive paper when viewing the canopy from the front. The placement of the water-sensitive paper in the depth direction of the canopy was positioned at the cross-sectional area where the air exits half of the canopy, as shown in Figure 12.

The arrangement of the water-sensitive paper within the canopy is shown in Figure 12a. Two sheets of water-sensitive paper are overlapped with their coated sides facing outwards (Figure 12b). They are placed in the yellow area and secured by paper clips, hanging tightly along the positioning line. The water-sensitive paper hangs vertically and parallel to the direction of the sprayer’s movement, ensuring that it can effectively collect data on the deposition of spray droplets after passing through the canopy. Additionally, the water-sensitive paper is suspended by a paper clip on the positioning line, allowing it to freely swing under the influence of air without any restrictions.

### 3.3. Air-Assisted Sprayer

**Figure 13:**
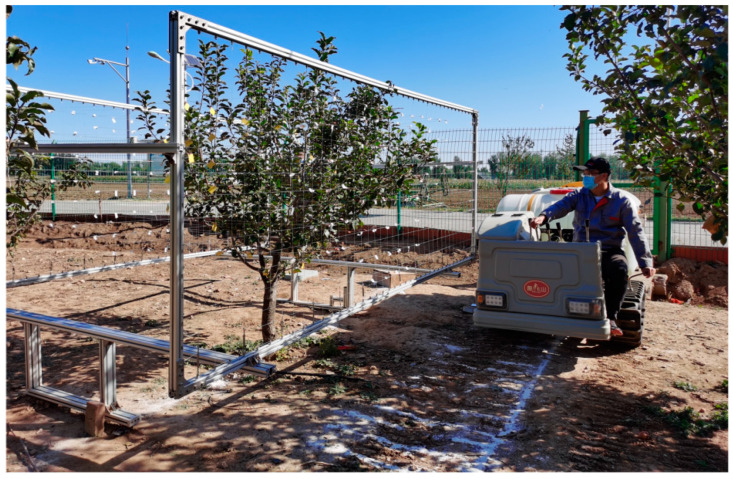
Orchard test of sprayer.

This study employed the Wolf Mountain 3WGF-300D air-assisted sprayer (Figure 13) for pesticide application. The working pressure of the sprayer was set between 1.2 MPa and 1.5 MPa, with a pump flow rate of 60 L/min, fan speed ranging from 0 to 2800 r/min, and a tank capacity of 300 L. The dimensions of the sprayer were 2.5 m × 1.3 m × 1.16 m, with a spraying width greater than 20 m and a spraying height approximately greater than 7 m. The operational speed ranged from 3 km/h to 4.2 km/h.

During the experiment, the spraying volume was set at 40 L/min. The working speed was set at 3 km/h, meeting the requirement mentioned in the article by Zhai et al. (2018) [3] that the operational speed of the sprayer should be less than 5.4 km/h. The sprayer moved along the tree rows while spraying, and both side nozzles were opened for spraying, to closely simulate actual field operations.

### 3.4. Experiment Methods

LiDAR sensors can emit and receive high-frequency laser signals, calculating distances through the time-of-flight principle. The laser beam of the LiDAR sensor can be obtained by the varying scanning angle and reflected time. The characteristics of the target canopy can then be obtained by processing the point cloud [22,32,33].

The orchard tree canopy was divided into grids of 0.2 m × 0.2 m using the grid partitioning method, combined with the air supplied width of the sprayer, which had been demonstrated through the range of the air delivery width of the sprayer experiment [8]. By processing the point cloud data [29], geometric parameters such as thickness, height, and width of different positions of the canopy were obtained. Through the method of CMPC [29], the canopy volume, thickness and leaf area in different areas of the canopy were calculated.The principle CMPC method is a grid-based map of the tree canopy. The grid size has a constant value of 0.2 m × 0.2 m. The key influencing factor of canopy volume is canopy thickness. Therefore, canopy thickness was chosen instead of volume in the study. This can be more direct and will not affect the experimental results. The air speed of the inlet and the outlet of the canopy was measured through wind loss model experimental studies.

During the experiment, the sprayer moved along the orchard tree rows at the set speed and spray volume. The sprayer was operated with spraying before reaching the canopy to achieve a stable working state. After each operation, the water-sensitive paper was collected sequentially after it dried completely. The spray fan speed was adjusted for experiments on droplet deposition under different fan speeds.

### 3.5. Data Analysis of Water-Sensitive Paper

In the experiment, water-sensitive paper produced by Chongqing Liuliu Mountain Plant Protection Technology Co., Ltd. was used to obtain droplet deposition data. The Liuliu Mountain Droplet Analysis software 1.0 was employed for processing, which facilitated the extraction of droplet parameters from the water-sensitive paper. The relevant parameters describing droplet deposition effectiveness on the water-sensitive paper included droplet density, droplet volume median diameter, and coverage. Prior to data analysis, the water-sensitive paper underwent preprocessing. It was scanned using a scanner to obtain grayscale images, which were saved in JPG format. The scanned images were then imported into the droplet analysis software. The software enabled selecting the analysis region, extracting the analysis region, adjusting foreground and background pixels, separating foreground from background, deselecting droplets, and excluding areas from analysis, facilitating the processing of water-sensitive paper parameters and statistical analysis of the results.

Droplet analysis software provided deposition data for droplets within the canopy, primarily including DV1, VMD, DV9, NMD, spectrum, total, deposits/cm^2^, coverage, area, and μL/cm^3^. The meanings of these symbols are shown in Table 10.

The study investigates the impact of canopy inlet air speed, thickness, and leaf area on the deposition of droplets on water-sensitive paper, aiming to obtain water-sensitive paper that effectively captures the deposition of droplets during the experimental process. The criterion for selecting the indicator is that the droplets should not aggregate into flow and slide down on the water-sensitive paper, and there should be no overlap between droplets.

The study of droplet deposition on water-sensitive paper is conducted through the following three steps:

(1) Calculation: During the deposition process of droplets on water-sensitive paper, when the coverage of droplets on the paper is significant (Figure 3), and there is excessive deposition leading to the washing away of droplets from the paper, it becomes difficult to accurately determine the deposition situation. Based on the results obtained from Section 2.1, the water-sensitive paper data under such conditions are excluded using the VMD (visual maximum droplet) value as the criterion.

(2) Standardization: According to the criteria specified in the Chinese standard GB/T 17997-1999 for coverage evaluation [34], when using low-volume spraying, the droplet density on the crop leaf area should be equal to or greater than 25 droplets/cm^2^ to achieve effective deposition; for disease control purposes, the droplet density on the crop should be equal to or greater than 70 droplets/cm^2^ to achieve effective coverage. Since the primary goal in routine maintenance of orchard trees is disease prevention, water-sensitive paper data with droplet densities of 25 droplets/cm^2^ or higher are extracted for further analysis.

(3) Exclusion: By observing the deposition of droplets on the water-sensitive paper, papers with evident clustering and flow of droplets are excluded. Papers with aggregated droplets forming a flow and flowing away from the water-sensitive paper cannot accurately reflect the actual deposition of droplets at that location. The deposition analysis obtained through software will underestimate the actual spray volume during the spraying process.

## 4. Discussion

Based on the study of the VMD, it is found that during the operation of the sprayer, when the outlet area remains constant, the higher the fan speed, the greater the air speed generated by the sprayer. However, the air speed does not continuously increase. After reaching a certain value, the air speed will decrease. When the fan speed exceeds this value, the air speed will continue to increase. It is not true that a higher fan speed results in better deposition of droplets within the canopy. If the air speed is too high, the VMD of droplet deposition within the canopy increases, and there is a significant presence of large droplets and droplet aggregation on the water-sensitive paper, leading to more pronounced pesticide drift.

The influence of canopy inlet air speed, canopy thickness, and leaf area on the total deposition and deposition ratio of droplets on both sides of the water-sensitive paper is relatively weak. Their impact on the model’s significance is consistent, with the order of impact from greatest to smallest being inlet air speed, leaf area, and canopy thickness. The influence of leaf area on the spray effect is not significant due to the sparse distribution of tree leaves within the selected orchard tree canopy in the study, along with a thick canopy and low leaf density. The air speed disturbance causes changes in leaf inclination, reducing the impact of the leaves on droplet deposition. During the spraying process, the initial velocity of droplets is relatively high due to the pressure in the pipeline, making it easier for droplets to penetrate the canopy, rendering the effect of air speed on droplet deposition insignificant. Under the dual influence of the initial droplet velocity and air speed, the effect of canopy thickness on droplet deposition is also not significant.

In the study, a constant spray volume was used for the sprayer, and there was no investigation of different spray volumes under different airflow conditions. The spray rate range was not specified, and the range of fan air speed was small. In future research, it is necessary to study different spray rates under low and high air speeds, design reasonable experimental plans, and conduct research on different periods and varieties of orchard trees in order to provide reasonable ranges of air speed and spray rates for variable-rate spraying.

## 5. Conclusions

Precision pesticide application technology based on volume detection can result in varied application effectiveness across different density areas of orchard trees. Wind speed, canopy volume, and leaf area within the canopy are crucial factors affecting application effectiveness. This study on application effectiveness based on the above comprehensive factors can provide a variable basis for precise regulation of both wind force and pesticide dosage, enabling their coordinated control. This manuscript studied the deposition effect of droplets within the canopy under different air speed conditions of the sprayer. Analyzed the VMD of deposited droplets, the total deposition within the canopy, and the ratio of deposition on both sides of the water-sensitive paper under different experimental conditions.

The study investigated the correlation between the coverage rate of droplets on the water-sensitive paper and the deposition quantity. It was found that they had a significant quadratic correlation. Either indicator can be used for research analysis. The VMD was identified as the measure of droplet deposition on the water-sensitive paper. The analysis revealed that areas with abnormal VMD values had low air loss rates, sparse distribution of canopy leaves, and smaller leaf area values. The study also examined the influence of different fan speeds on the inlet air speed within the canopy. It was found that the inlet air speed does not necessarily increase with the increasing fan speed of the sprayer. The lowest inlet air speed corresponds to a fan speed of 1502 r/min, followed by 1381 r/min, and the highest inlet air speed corresponds to a fan speed of 1676 r/min. The air speed varied greatly in locations with sparse canopy leaves.

The paper proposed combining the deposition quantities on both sides of the water-sensitive paper as the result of deposition for one side in bilateral spraying. The study found no correlation between canopy inlet air speed, canopy thickness, and leaf area with the total deposition. Through linear, quadratic, and cubic analysis, it was determined that the order of impact of these three factors on total deposition is inlet air speed, leaf area, and canopy thickness. Additionally, the study examined the influence of canopy inlet air speed, canopy thickness, and leaf area on the deposition ratio (ratio of deposition on both sides of the paper). It was observed that the correlation between these factors and the deposition ratio was weak. The linear, quadratic, and cubic analysis of these factors with the deposition ratio revealed that the order of impact from large to smallest is inlet air speed, leaf area, and canopy thickness. Multiple regression analysis was conducted to obtain regression models for the above three factors with both the total deposition and the deposition ratio. However, the models had low precision in fitting the data, indicating the need for optimization of experimental methods and further research.

## Figures and Tables

**Table 1:** Locations with smaller VMD values correspond to canopy features and input airflow speed conditions.

Water-Sensitive Paper Position	Canopy Inlet Air Speed (r/min)	Air Speed Loss Rate (%)	Canopy Thickness (m)	Canopy Leaf Area (cm^2^)
2-2	3.01	70.8	0.7	2696
3-6	2.1	93.7	1.61	1562
5-2	7.79	83.7	1.25	2672
7-6	5.3	89.8	1.87	1173

**Table 2:** Locations with larger VMD values (Figure 7a) correspond to canopy features and input airflow speed conditions.

Water-Sensitive Paper Position	Canopy Inlet Air Speed (r/min)	Air Speed Loss Rate (%)	Canopy Thickness (m)	Canopy Leaf Area (cm^2^)
1-2	3.937	56.3	0.155	800
1-3	2.593	47.3	0.265	837
3-3	3.53	70.7	1.185	3801
10-2	3.08	18.2	0.495	1229
10-3	2.63	14.1	0.395	1333
11-1	3.277	73.1	0.545	184
12-1	3.12	30.9	0.63	188
4-6	4.133	80.5	1.38	1814

**Table 3:** Correlation between inlet air speed, canopy thickness, leaf area, and total droplet deposition.

Influence Factor	Inlet Airflow Speed	Canopy Thickness	Leaf Area
Canopy thickness	0.191		
Leaf area	0.424	0.155	
Total droplet deposition	0.045	0.128	0.023

**Table 4:** R^2^ value of the fitting equation among inlet airflow speed, canopy thickness, leaf area, and total droplet deposition.

Fit Times	Inlet Air Speeds	Canopy Thickness	Leaf Area
Linear	0.002	0.004	0.016
Quadratic	0.032	0.026	0.028
Cubic	0.05	0.026	0.033

**Table 5:** *p* value of the fitting equation among inlet airflow speed, canopy thickness, leaf area, and total droplet deposition.

Fit Times	Inlet Air Speeds	Canopy Thickness	Leaf Area
Linear	0.630	0.533	0.152
Quadratic	0.062	0.091	0.247
Cubic	0.142	0.950	0.473

**Table 6:** Fitting equation analysis of variance for inlet airflow speed, canopy thickness, leaf area, and total droplet deposition.

Source	Degree of Freedom	Adj SS	F Value	*p* Value	R^2^
Regression	5	241.19	0.83	0.531	0.004
C_1_	1	12.51	0.22	0.644	
C_2_	1	11.63	0.20	0.655	
C_3_	1	16.50	0.28	0.595	
C_1_* C_1_	1	37.18	0.64	0.426	
C_2_* C_2_	1	41.75	0.72	0.399	

**Table 7:** R^2^ value of the fitting equation among inlet air speed, canopy thickness, leaf area, and total deposition ratio.

Fit Times	Inlet Air Speeds	Canopy Thickness	Leaf Area
Linear	0.005	0.003	0.014
Quadratic	0.018	0.014	0.016
Cubic	0.036	0.019	0.014

**Table 8:** *p* value of the fitting equation among inlet air speed, canopy thickness, leaf area, and total deposition ratio.

Fit Times	Inlet Air Speeds	Canopy Thickness	Leaf Area
Linear	0.452	0.599	0.217
Quadratic	0.225	0.260	0.562
Cubic	0.145	0.391	0.102

**Table 9:** Fitting equation analysis of variance for inlet air speed, canopy thickness, leaf area, and total deposition ratio.

Source	Degree of Freedom	Adj SS	F Value	*p* Value	R^2^
Regression	8	1,398,736	0.64	0.744	0.004
C_1_	1	153,607	0.56	0.455	
C_2_	1	270,269	0.99	0.323	
C_3_	1	205,047	0.75	0.389	
C_1_* C_1_	1	167,591	0.61	0.436	
C_2_* C_2_	1	285,699	1.04	0.309	
C_3_* C_3_	1	76,589	0.28	0.598	
C_2_* C_2_* C_2_	1	158,836	0.58	0.448	
C_3_* C_3_* C_3_	1	47,840	0.17	0.677	

**Table 10:** Meaning of data parameters obtained by droplets analysis software.

Noun	Interpretation
DV1	The number of sampled droplets was accumulated in order of increasing droplet volume, with the cumulative value corresponding to the droplet diameter that represents 10% of the total number of sampled droplets, measured in micrometers (μm).
VMD	Volume median diameter refers to the droplet diameter that corresponds to the accumulation of droplets in order of increasing volume, with the cumulative value equal to 50% of the total number of sampled droplets, measured in micrometers (μm).
DV9	The number of sampled droplets was accumulated in order of increasing droplet volume, where the cumulative value corresponds to the droplet diameter that represents 90% of the total number of sampled droplets, measured in micrometers (μm).
NMD	Quantity median diameter: The number of sampled droplets was accumulated in order of increasing droplet size, where the cumulative value corresponds to the droplet diameter that represents 50% of the total number of sampled droplets, measured in micrometers (μm).
Spectrum	Droplet spectrum width: The condition of the size distribution of droplet diameters, which measures the uniformity of droplet sizes.
Total	The number of droplets within the selected region.
Deposits/cm^2^	Droplet density.
Coverage	Coverage rate.
Area	The area of the selected region.
μL/cm^3^	Pesticide deposition.

## Data Availability

The raw data supporting the conclusions of this article will be made available by the authors, without undue reservation.
